# Enhancement of Anti-Inflammatory Activity of Optimized Niosomal Colchicine Loaded into Jojoba Oil-Based Emulgel Using Response Surface Methodology

**DOI:** 10.3390/gels8010016

**Published:** 2021-12-25

**Authors:** Heba S. Elsewedy, Nancy S. Younis, Tamer M. Shehata, Maged E. Mohamed, Wafaa E. Soliman

**Affiliations:** 1 Department of Pharmaceutical Sciences, College of Clinical Pharmacy, King Faisal University, Alhofuf 36362, Saudi Arabia; nyounis@kfu.edu.sa (N.S.Y.); tshehata@kfu.edu.sa (T.M.S.); Memohamed@kfu.edu.sa (M.E.M.); 2 Department of Pharmaceutics, College of Pharmacy, Zagazig University, Zagazig 44519, Egypt; 3 Department of Pharmacognosy, College of Pharmacy, Zagazig University, Zagazig 44519, Egypt; 4 Department of Biomedical Sciences, College of Clinical Pharmacy, King Faisal University, Alhofuf 36362, Saudi Arabia; weahmed@kfu.edu.sa; 5 Department of Microbiology and Immunology, Faculty of Pharmacy, Delta University for Science and Technology, Gamasa, Mansoura 11152, Egypt

**Keywords:** anti-inflammatory, colchicine, niosome, emulgel, optimization

## Abstract

Recent progression in investigational studies aiming to integrate natural products and plant oils in developing new dosage forms that would provide optimal therapeutic effect. Therefore, the aim of the present exploration was to inspect the influence of jojoba oil in boosting the anti-inflammatory effect of colchicine natural product. To our knowledge, there is no formulation comprising colchicine and jojoba oil together to form a niosomal emulgel preparation anticipated for topical application. Colchicine is a natural product extracted from *Colchicum autumnale* that has been evidenced to show respectable anti-inflammatory activity. Owing to its drawbacks and low therapeutic index, it was preferable to be formulated into topical dosage form. The current study inspected colchicine transdermal delivery by developing niosomal preparation as a potential nanocarrier included into emulgel prepared with jojoba oil. Box Behnken design was constructed to develop 17 niosomal emulgel formulations. The optimized colchicine niosomal emulgel was evaluated for its physical characteristics and in vitro release studies. The in vivo anti-inflammatory activity was estimated via carrageenan-induced rat hind paw edema method. The developed colchicine niosomal preparation revealed particle size of 220.7 nm with PDI value 0.22, entrapment efficiency 65.3%. The formulation was found to be stable showing no significant difference in particle size and entrapment efficiency up on storage at 4 °C and 25 °C for 3 months. The optimized colchicine niosomal emulgel exhibited a pH value 6.73, viscosity 4598 cP, and spreadability 38.3 mm. In vitro release study of colchicine from niosomal emulgel formulation was around 52.4% over 6 h. Apparently, the proficient anti-inflammatory activity of colchicine niosomal emulgel was confirmed via carrageenan-induced rat hind paw edema test. Overall, the results recommend the combination of niosomal preparation with jojoba oil-based emulgel that might signify a favorable delivery of anti-inflammatory drug such as colchicine.

## 1. Introduction

Utilizing nanotechnology in the field of drug delivery is an advanced way to deliver drugs on a nanoscale to specific organs in a controlled manner to maximize their therapeutic action and reduce the side effects [1]. Various nanocarriers have been developed for entrapping several drugs and intended to be delivered to specific tissues such as nanoemulsion, nanoparticles, liposome, niosome, and others [2]. Niosomes are regarded as one of the nanocarriers that support the delivery, targeting, and controlled release of drugs. Further, niosome exhibits a great impact in delivering naturally occurring pharmaceutical substances in addition to enhancing their physical stability and efficacy [3]. They are principally self-assembled bilayer vesicles formed of nonionic surfactants and cholesterol [4]. It has the benefit of encapsulating and delivering both hydrophilic and hydrophobic drugs. It could be transported by various routes of delivery including oral, parenteral, and topical ways [5].

Topical drug delivery is a way of applying drug to specific area on the skin, however; penetration of the drug could be a critical matter. Thus, certain vesicular systems such as niosomes, liposomes, and proniosomes have been facilitated for such purpose. Niosomes have been considered as candidate nanocarrier for topical drug delivery owing to their advantages in improving the drug penetration and drug release [6]. Additionally, niosomes are considered more stable than other systems including liposome as a result of non-ionic surfactant [7].

Certain difficulties may arise while applying niosomal formulation topically owing to its low viscosity that gives rise to inappropriate skin application [8]. Thus, incorporating niosome into emulgel formulation would feasibly increase the formulation viscosity and consequently, support its topical application [9]. Emulgel has been identified as a transdermal drug-delivery system composed of gel and emulsion used as a vehicle to deliver the drug [10]. Emulgel has the ability to enhance skin permeability and accordingly augment drug efficacy [11]. It is better for the delivery of anti-fungal, antibacterial, analgesics, and anti-inflammatory drugs [12].

At present, medicinal plant oils are characterized by their cheapness, availability, and effectiveness. Jojoba oil is one of these vegetable oils, being a liquid extracted from seeds of *Simmondsia chinensis* plant [13]. It has been revealed to be excellent at improving topical drug absorption [14,15]. It was confirmed to play a potential role in treating skin diseases, such as acne, dermatitis, and eczema [16]. It could be involved widely in moisturizers, sunscreen, and cosmetic preparations in addition to its evidenced action as anti-inflammatory drug [17]. Jojoba oil has been included within a number of nanocarriers in order to obtain various delivery systems, namely; microemulsion, nanoemulsion [18], emulsion, and emulgel [19].

Regarding natural products, they have been extensively applied for treating various disorders due to their considerable pharmacological potential and safety [20]. Colchicine is renowned as a natural alkaloid derived from *Colchicum autumnale* plant. It is considered being the drug of choice in treating and preventing acute gout [21]. Recently, colchicine has been verified to target several mechanisms concomitant with COVID-19 excessive inflammation [22]. It has been proven to have cardio-protective effect [23] and anti-inflammatory action against rheumatoid diseases [24,25]. In the light of that, integrating niosomal preparation loaded with colchicine into jojoba oil-based emulgel possibly would ameliorate the efficiency of the drug and enable their topical delivery.

To ensure the quality of the formulation, an organized and systematic approach known as response surface methodology (RSM) should be applied. It depends on the influence of certain variables on observed responses to get the optimized formula with optimal features [26]. RSM has several mathematical designs; the most frequently used of them is Box Behnken Design (BBD). It is a software design that requires three levels for each factor with fewer numbers of runs, providing mathematical equations along with some graphical models to understand the relation between variables and responses [27].

In these perceptions, the objective of current investigation was to develop colchicine-loaded niosome incorporated into jojoba oil-based emulgel optimized via employing a 3^3^ full factorial design and demonstrate the potential of the optimized niosomal emulgel formulation as topical anti-inflammatory agents.

## 2. Results and Discussion

Based on our preliminary studies, a dispersion of niosome was successfully developed with an optimum molar ratio of nonionic surfactant and cholesterol 1:1 in order to attain a physically stable niosome.

### 2.1. Entrapment Efficiency

Entrapment efficiency of developed colchicine loaded niosome was successfully assessed using centrifugation method. The higher solubility of colchicine in water and in surfactant/cholesterol lipid mixture provides a high entrapment efficiency of colchicine, that being 65.3 ± 2.2%. Actually, the type of non-ionic surfactant plays a vital role in the percentage of drug that could be entrapped. Span 60 possesses high transition temperature (53 °C), which participates in higher entrapment efficiency [28]. Moreover, collaboration of cholesterol and non-ionic surfactant stimulates the increase in thickness and surface area of the niosomal layers in addition to the rigidization caused by the surfactant, which results in high entrapment [29]. Further, the molar ratio of cholesterol to non-ionic surfactant participates in achieving higher entrapment into the niosomes [30]. Previous literature stated that colchicine was highly entrapped into niosomes upon adjusting the molar ratio to be 1:1 between surfactant and cholesterol [31].

### 2.2. Vesicular Size and Size Distribution (PDI)

**Figure 1:**
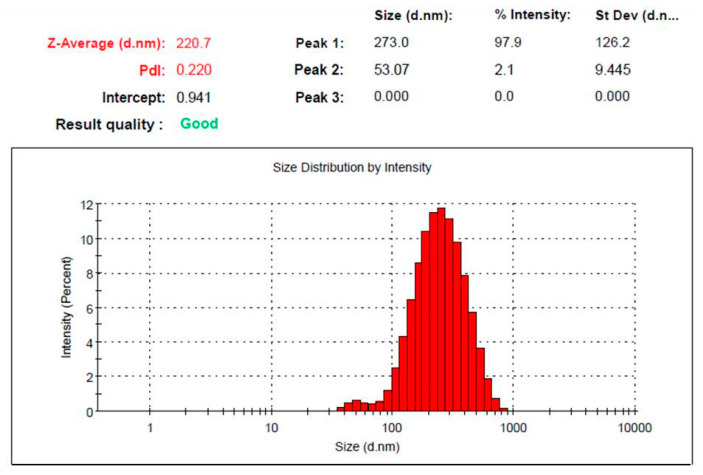
Vesicular size and size distribution curve of colchicine loaded niosome.

Particle size and PDI measurement of the developed colchicine-loaded niosome were estimated and data displayed in Figure 1. It exhibited particle size of 220.7 ± 2.3 nm, with good distribution (PDI 0.22), which indicate that the particles fall within a narrow range of sizes.

### 2.3. Stability Study

**Figure 2:**
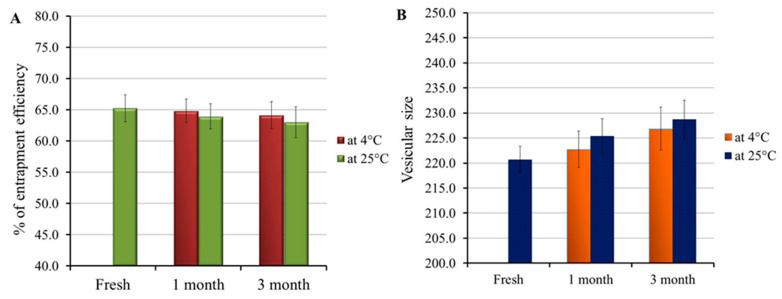
Stability study outline for colchicine loaded niosome formulation investigated at 4 °C and 25 °C for 1 and 3 months in terms of (**A**) vesicular size and (**B**) entrapment efficiency, compared to freshly prepared formulation.

Examining the stability of the developed colchicine-loaded niosome was accomplished and the results are depicted in Figure 2A,B. Non-significant variations were distinguished in the entrapment efficiency and vesicular size of the formulation following 1 and 3 months storage at 4 °C and 25 °C if compared to freshly prepared niosomal preparation (*p* < 0.05). The study outcomes emphasized the formulation stability and support niosome for a potent drug carrier. Actually, presence of DSPE-PEG represents a key role in increasing formulation stability, since it offers certain steric hindrances for the formulation membrane [32,33].

### 2.4. Experimental Design

#### 2.4.1. Fitting the Model

A matrix of 17 runs was generated upon constructing BBD software. The effect of each independent variable on the investigated dependent response of the developed niosomal emulgel formulations is obvious in Table 1.

#### 2.4.2. Statistical Analysis of the Design

Analysis of variance for all the dependent variables was carried out using BBD software and certain parameters including *p*-value, F-value, and model F-value were obtained using ANOVA. Primarily, the best model to fit for all responses was the quadratic one if compared to all other models. Referring to Table 2, it was noticed that *p*-value for all responses were less than 0.0001, which is necessary for confirming that the independent variables showed a significant effect on the investigated response. Relating to F-value of the responses, it was proven to be greater values which are recommended to provide less error in the model. The model F-value for R_1_ and R_2_ is 67.64 and 35.53, respectively, which implies that the model is significant. Regarding lack of fit, it is mandatory to be non-significant in order to fit the model and confirm its efficiency [34]. It was apparent that lack of fit was 2.18 and 2.20, which is not significant relative to the pure error in addition to their related *p*-value 0.2333 and 0.2304 for R_1_ and R_2,_ respectively, which indicates that the chance for this large value due to noise was 23.33 and 23.04%, respectively.

### 2.5. Effect of Independent Variables on Viscosity (R_1_)

Viscosity is a very important parameter for topical preparations, since it may alter the diffusion of the drug and consequently affect its in vitro release [35]. The viscosity of various formulated colchicine niosomal emulgel was measured and data are summarized in Table 1. The formulations viscosity was extended between 4265 ± 184 and 9834 ± 210 cP. It was highly obvious that a parallel relation was detected between the viscosity of all niosomal emulgel preparations and the concentration of the different independent variables used. Accordingly, upon increasing X_1_, X_2_, and X_3_, a consistent increase in formulations viscosity was observed and this is certainly due to the formulation ingredients [36]. In consequence, greater oil concentration in the formulation contributed to an increment in viscosity. As well, higher concentration of gelling agent gave rise to corresponding increase in formulation viscosity [10]. Regarding the influence of X_2_ on the R_1_ response, it was revealed that upon using the same concentration of oil and gelling agent, an increase in niosomal emulgel was confirmed. This noticeable impact of the three factors on the viscosity was further illustrated by the following mathematical equation:

R1 = 6229.6 + 2124.12 X_1_ + 181.125 X_2_ + 673.5 X_3_ + 264.25 X_1_X_2_ + 319 X_1_X_3_ + 160 X_2_X_3_ + 585.325 X_1_^2^ − 6.675 X_2_^2^ − 84.425 X_3_^2^

**Figure 3:**
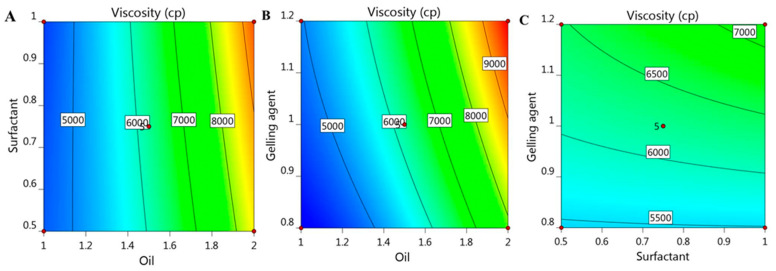
2D contour graph representing the effect of independent factors (**A**) X_1_ and X_2_, (**B**) X_1_ and X_3_, and (**C**) X_2_ and X_3_ on viscosity responses (R_1_).

**Figure 4:**
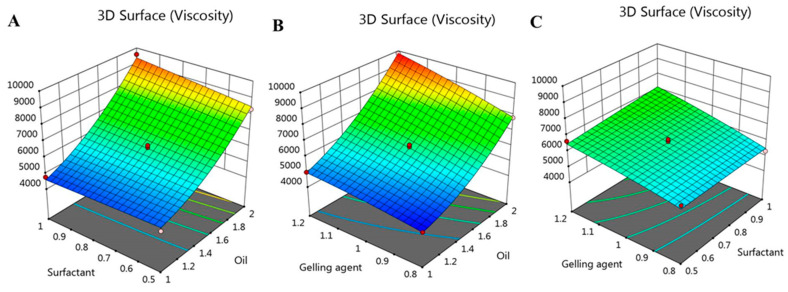
3D-response surface plot representing the effect of independent factors (**A**) X_1_ and X_2_, (**B**) X_1_ and X_3_, and (**C**) X_2_ and X_3_ on viscosity responses (R_1_).

As noted, the positive sign in the equation confirmed the synergistic effect of X_1_, X_2_, and X_3_ on R_1_ response. As well, the previous positive effect was emphasized and represented by 2D contour graph and 3D-response surface plot as portrayed in Figure 3 and Figure 4.

**Figure 5:**
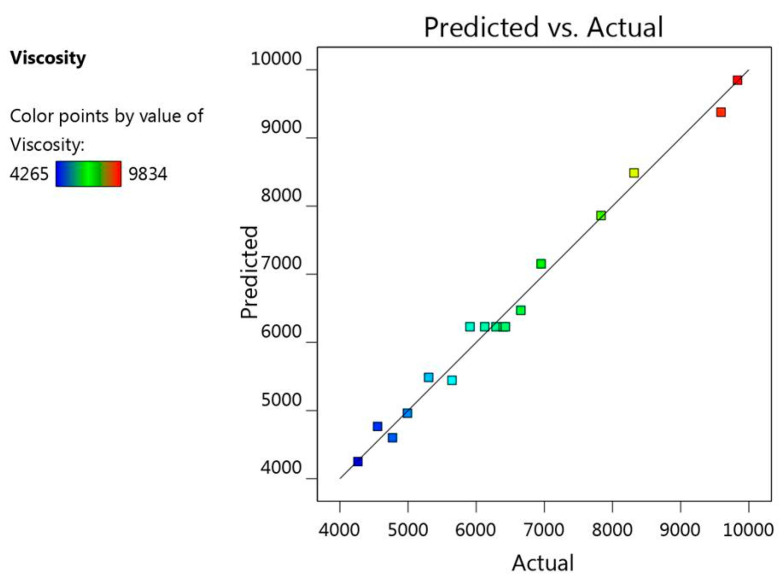
Linear correlation plot between actual and predicted values for viscosity response (R_1_).

Furthermore, there was a linear correlation detected between the predicted versus the actual responses where the predicted R^2^ for response R_1_ is (0.8805), which is in reasonable agreement with the adjusted R^2^ (0.9740) as illustrated in Table 2. Additionally, Figure 5 presented the residual values that were distributed among the two sides of the line signifying that the actual data and the predicted values were in a credible correlation with each other. Likewise, R^2^ value (0.9886) provides an indication that the system could recommend the model, adding to that the adequate precision (27.6989), which is a desirable value elucidating an adequate signal and concluding that the model could navigate the design space.

### 2.6. Effect of Independent Variables on In Vitro Release Study (R_2_)

**Figure 6:**
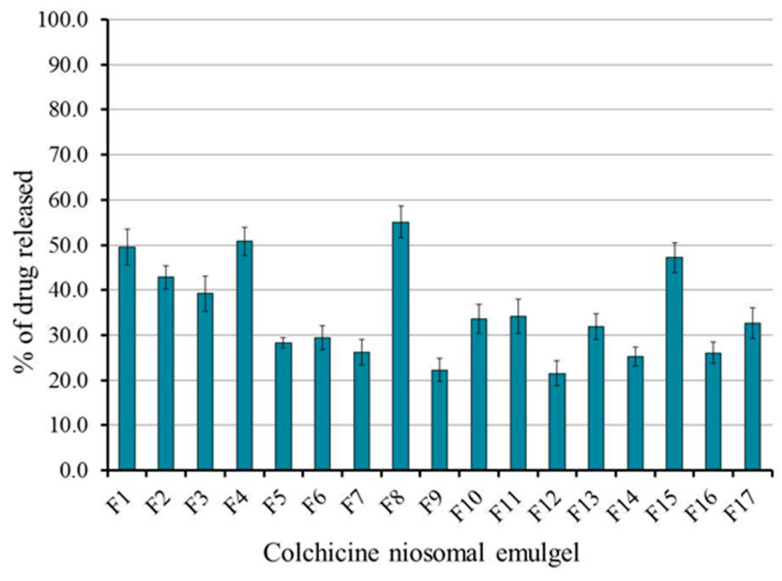
In vitro release of colchicine from different niosomal emulgel formulations maintained at 37 °C using phosphate buffer pH 7.4 following 6 h. Results are expressed as mean ± SD of three experiments.

**Figure 7:**
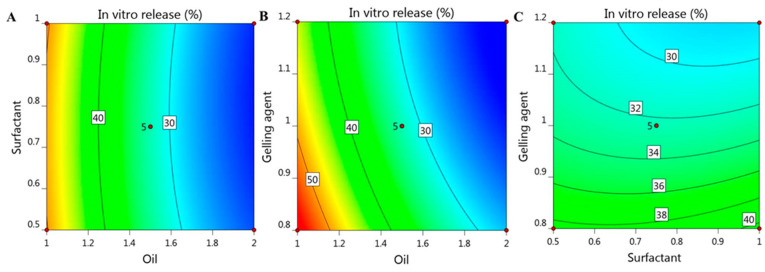
2D contour graph representing the effect of independent factors (**A**) X_1_ and X_2_, (**B**) X_1_ and X_3_, and (**C**) X_2_ and X_3_ on in vitro release response (R_2_).

**Figure 8:**
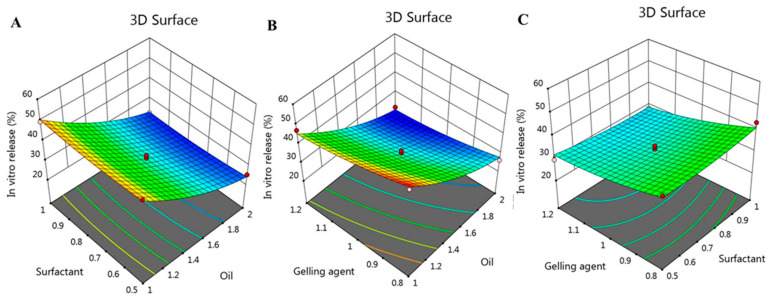
3D-response surface plot representing the effect of independent factors (**A**) X_1_ and X_2_, (**B**) X_1_ and X_3_, and (**C**) X_2_ and X_3_ on in vitro release response (R_2_).

The features of the in vitro release study of colchicine from the prepared niosomal emulgel formulations were competently assessed and the outcome was outlined in Figure 6. The investigation was prolonged for 6 h to achieve percentages of drug release that ranged from 21.5 ± 2.7 to 55.1 ± 3.6. It was remarkable that the percentage of colchicine released from all formulations was affected greatly by the examined independent variables. Generally, increasing oil concentration would cause retardation in the process of drug release from the formulation [37]. Additionally, a decrease in the drug release usually perceived upon increasing the concentration of the gelling agent [38]. In the current study, a considerable decrease in the in vitro release of colchicine from the developed niosomal emulgel formulations was observed while increasing the concentration of all independent factors X_1_, X_2_, and X_3_. The variations in the drug release percentage could be correlated to the formulation viscosity, since higher viscosity can produce resistance to drug dispersion and movement and slow the rate of drug dissolution [39]. In view of that, niosomal emulgel formed of higher concentrations of oil, surfactant, and gelling agent exhibited higher viscosity and consequently showed minor percentage of drug release [36]. The next regression equation interprets the previously stated influence of the independent variables X_1_, X_2_, and X_3_ on the in vitro release response R_2_:

R_2_ = 52.36 − 13.4375 X_1_ − 0.2375 X_2_ − 4.6 X_3_ − 0.4 X_1_X_2_ + 0.825 X_2_X_3_ − 1.225 X_1_X_3_ + 3.57 X_1_^2^ + 1.02 X_2_^2^ + 1.545 X_3_^2^

besides, the interactions between the independent variables and the examined response (R2) was further clarified by the model graphs as illustrated in Figure 7 showing 2D contour graph and Figure 8 displaying 3D surface plot.

**Figure 9:**
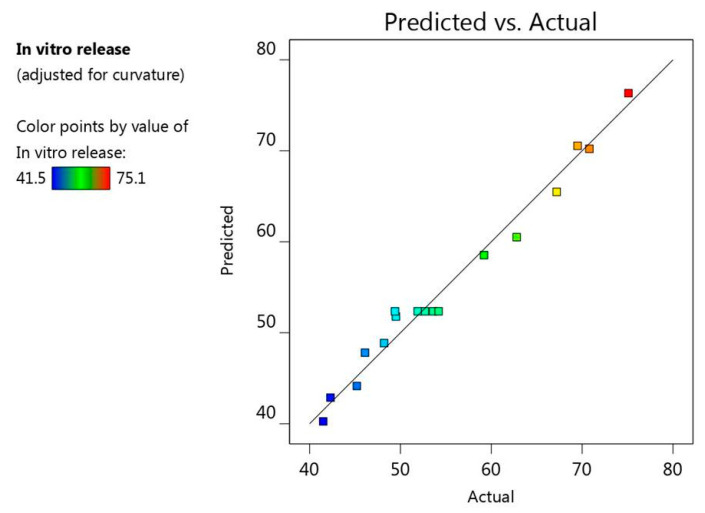
Linear correlation plot between actual and predicted values for in vitro release response (R2).

On the other track, and as revealed from Figure 9, there was a linear relationship between the predicted and the actual responses, since the predicted R^2^ (0.7739) was found to be in a sensible harmony with the adjusted one (0.9510) because the difference is less than 0.2 as demonstrated in Table 2. As well, the value of R^2^ (0.9786) indicates that the system could support the model and the adequate precision value (20.4175), which is a recommended value proving that the model could navigate the design space.

### 2.7. Optimizing the Investigated Variables

In order to provide the optimal characteristics and appropriate levels of constraints that lead to the utmost value of desirability, an optimization technique was executed [40]. A numerical optimization was conducted by the design software next to constructing various graphs that were produced to illustrate the interaction between the independent variables. The optimization process depends on pointing the responses toward certain criteria that are expected to offer the optimized formula. The selected criteria in our study were to minimize the vesicular size and maximize the in vitro release. Taking into account the post analysis data that were provided in the point prediction, the expected actual values of independent variables were (1.04 g) jojoba oil, (0.9 g) Tween 80, and (0.803 g) Na alginate. The anticipated values for the responses based on higher desirability were 4187.4 cP for R_1_ and 55.53% for R_2_. With regard to the previous predictions, the optimized niosomal emulgel formulation was developed and its obtained responses were matched with the predicted values and as per the data in Table 3, both values were prominently close to each other.

### 2.8. Characterization of Optimized Topical Formulations

The optimized colchicine niosomal emulgel was studied for its characteristics and compared to the conventional colchicine niosomal gel and data are presented in Table 4. Both formulations were homogeneous and showed acceptable physical appearance. The pH is in an appropriate range that insures no skin sensitivity or irritation. For the formulations viscosity, it was assessed and showed that incorporating jojoba oil in niosomal emulgel would result in higher viscosity than niosomal gel; however, viscosity of both preparations being in a good range that could be easily applied topically. As for spreadability, it was measured and their values confirmed that the formulations could spread evenly over the skin. Comprehensively, despite the significant difference that was observed between the formulations in certain parameters, nevertheless, the estimated features of niosomal gel and emulgel incorporating colchicine appear to be satisfactory and appropriate for skin application.

### 2.9. Assessment of In Vitro Drug Release Studies

**Figure 10:**
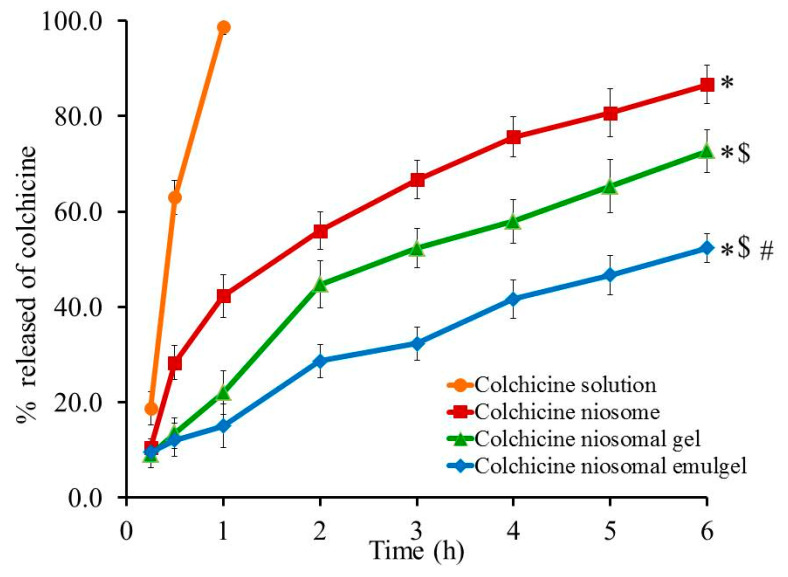
In vitro release study of colchicine from different formulations compared to colchicine solution in phosphate buffer pH 7.4 at 37 °C. Results are expressed as the mean ± SD of three experiments. * *p* < 0.05 compared to colchicine solution; $ *p* < 0.05 compared to colchicine niosome and # *p* < 0.05 compared to colchicine niosomal gel.

The percentage of colchicine released from different developed formulations and from colchicine solution across cellophane membranes was performed via Franz diffusion apparatus and results are shown in Figure 10. Almost 98.7% of colchicine was released from colchicine solution within one hour due to higher solubility of the drug. Conversely, it was noticed that 52.4 ± 3.0, 72.67 ± 4.5, and 86.67 ± 4.04% of colchicine was released from colchicine niosomal emulgel, colchicine niosomal gel, and colchicine niosome after 6 h, respectively. The significant reduction (*p* < 0.05) in the drug release from all formulations under investigation compared to colchicine solution could be ascribed to the well-known fact that niosomal formulation has the ability to form cement layer in the spaces of the niosomal bilayer membrane so it will affect the drug diffusion and slow down its release [41]. Moreover, the viscosity of the preparations plays a crucial role in its in vitro behavior, whereas, certain ingredients like gelling agents provide higher viscosity for the formulation that would support drug retention and allow the release in a controlled pattern. The previous illustration could explain the significant reduce (*p* < 0.05) of colchicine release from niosomal gel and emulgel formulation compared to colchicine niosome itself, since the former formulations comprise gelling agent. On the other side, the significant difference (*p* < 0.05) in colchicine release from niosomal emulgel compared with niosomal gel is due to the incorporation of jojoba oil and tween 80 that provides further retention for drug release from emulgel formulation [39].

### 2.10. Release Kinetic Mechanism

**Figure 11:**
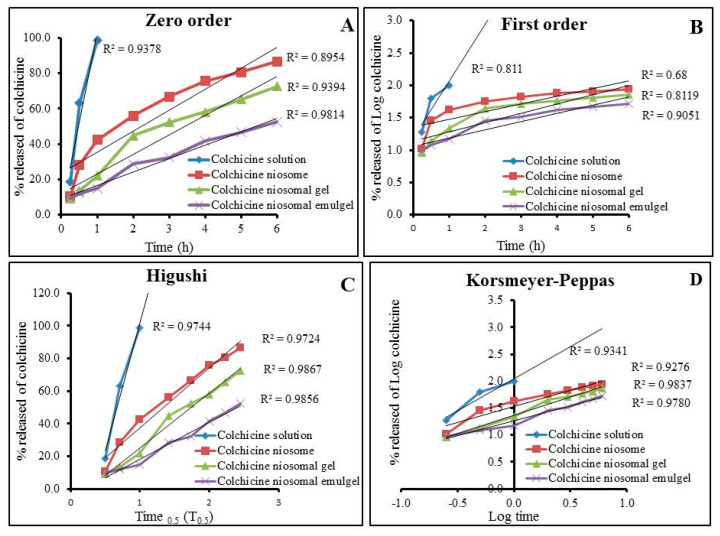
Percentage of drug released from developed Colchicine niosomal formulations against colchicine solution and their kinetic analysis according to (**A**) zero order, (**B**) first order, (**C**) Higauchi, and (**D**) Korsmeyer-Peppas model.

The kinetic analysis of all formulations under investigation was studied to show the mechanism by which the drug is released through the cellophane membranes and results are exhibited in Table 5 and Figure 11. Plotting the amount of drug released versus time elucidated that the release of colchicine from all formulations followed Higuchi model as it exhibited the most linear plot and established the greatest value of (r2). Higuchi model confirmed the drug has been released from matrix type with perfect sink conditions that were always achieved in the release environment [42]. Interestingly, the release of water-soluble drugs from several semi-solid formulations is extensively represented by Higuchi model [43].

### 2.11. Anti-Inflammatory Testing; Carrageenan-Induced Rat Paw Edema Test

**Figure 12:**
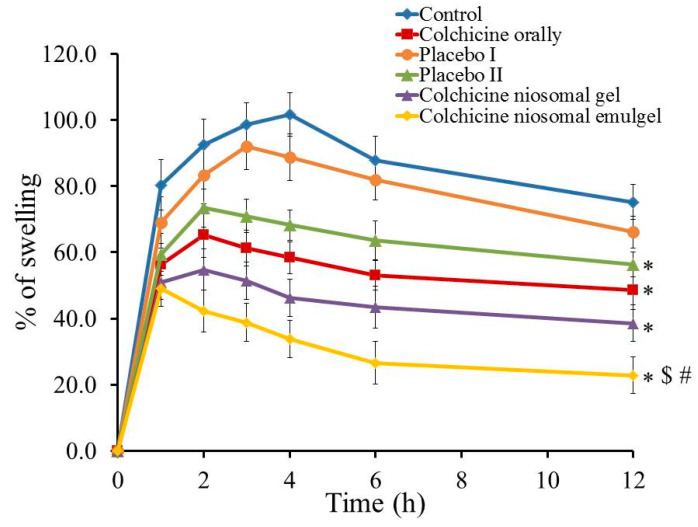
Effects of colchicine prepared in various formulations on percentage of swelling of carrageenan-induced paw edema rats. Results are expressed as mean with the bar showing SD (*n* = 5).* *p* < 0.05 compared to non-treated group (control); $ *p* < 0.05 compared to colchicine orally and # *p* < 0.05 compared to colchicine niosomal gel.

Figure 12 displays the percentage of swelling following topical application of colchicine niosomal gel and emulgel on carrageenan-induced rat hind paw edema compared to non-treated animal (control). The anti-inflammatory effect was calculated as percentage of swelling over time. The non-treated group depicted the utmost swelling after induction of inflammation after 4 h (101.6%). Likewise, the group treated with the placebo I formulation reached the maximum swelling following 3 h of starting the investigation achieving 92.0% and exhibiting a non-significant difference if compared to control-treated group (*p* < 0.05). On the other side, a significant reduction of swelling was obtained in placebo II, colchicine orally, colchicine niosomal gel and colchicine niosomal emulgel-treated groups if compared to control group (*p* < 0.05) where it exhibited 56.4, 48.6, 38.6, and 22.8% swelling, respectively, following 12 h. Incredibly, insignificant difference was observed between placebo II and colchicine orally treated group (*p* < 0.05), which proves the role of emulgel and mainly, jojoba oil in ameliorating the anti-inflammatory influence. In fact, the influence of jojoba oil in alleviating inflammation in different experimental models was previously established [44]. Prominently, animals treated with colchicine niosomal emulgel showed the most plentiful reduction in percentage of swelling, which is statistically significant (*p* < 0.05) comparing to all other groups treated with other applied preparations. Subsequently, colchicine niosomal emulgel provides the greatest anti-inflammatory potential. The present investigation offers strong confirmation about the improved permeation and enhanced effect of topical formulation incorporating jojoba oil and colchicine in niosomal emulgel and proposing a synergistic effect between them. The prospective of jojoba oil in lowering inflammation was confirmed earlier in our study that exhibited the synergism between jojoba oil and brucine as anti-inflammatory when incorporated into liposomal emulgel formulation [45].

## 3. Conclusions

Colchicine was successfully involved in niosomal preparation that is regarded as a promising drug delivery technique. For better application, the niosomal formulation was incorporated into various emulgel dosage forms. Box Behnken design approach was executed and came up with several niosomal emulgel formulas. The optimized of formula was selected based on values of certain investigated factors that was (1.04 g) jojoba oil, (0.9 g) Tween 80, and (0.803 g) Na alginate, and their obtained responses. The optimized formulation exhibited suitable physical properties including pH 6.73, viscosity 4598 cP, and spreadability 38.3 mm, in addition to an effective in vitro drug release study that reached 52.4% over 6 h. The developed formulations could mitigate hind paw inflammation induced by carrageenan since niosomal emulgel formulation containing jojoba oil could reduce paw swelling to reach 22.8%. In essence, the study revealed that the pharmacological action of colchicine was synergistically enhanced upon merging colchicine niosome with emulgel prepared with jojoba oil.

## 4. Materials and Methods

### 4.1. Materials

Colchicine was procured from Sigma-Aldrich Co. (St Louis, MO, USA). Poly ethylene glycol- distearoylphosphatidyl ethanolamine (DSPE-PEG 2000) was purchased from Lipoid LLC, (Newark, NJ, USA). Cholesterol, non-ionic surfactant (Sorbitan Monostearate; Span 60 and polysorbate 80; Tween 80), chloroform, and sodium alginate were purchased from Sigma-Aldrich Co. (St Louis, MO, USA). Jojoba oil was obtained from NOW^®^ Essential Oils (NOW Foods, Bloomingdale, IL, USA). All other solvents and chemicals of analytical grade were purchased from Sigma, USA.

### 4.2. Preparation of Colchicine Loaded Noisome

A method described previously by Shehata et al. was followed to formulate niosomal preparation using thin film hydration method [36]. Accordingly, non-ionic surfactant (span 60) and cholesterol with a molar ratio (1:1) were mixed together, followed by adding sufficient quantity of DSPE-PEG 2000 and then dissolved in chloroform using a round bottom flask. Afterward, under reduced pressure, the mixture was dried at 60 °C using rotary evaporator (Cole-Parmer, T-1602-21, Tokyo, Japan). A dried lipid film was formed on the wall of the flask, which was subjected to hydration using 5 mL dist. water containing colchicine (25 mg), while keeping agitation in water bath at 60 °C for 1 h to get niosomal solution. Consequently, the attained niosome was exposed to probe sonicator (XL-2000, Qsonica, Newtown, CT, USA) for 30 s to obtain the anticipated particle size.

### 4.3. Entrapment Efficiency

The entrapment efficiency of the developed colchicine-loaded niosome was considered as the percentage of colchicine carried by the preparations. Entrapment efficiency was assessed by employing centrifugation method. Niosomal formulation was added into Amicon^®^ ultra-4 (Ultracel-10K) and adjusted to be centrifuged at 6000 rpm using centrifuge (Andreas Hettich GmbH, Co.KG, Tuttlingen, Germany) while keeping the temperature at 4 °C for 1 h. The concentration of the free un-entrapped drug in the filtrate was measured spectrophotometrically at 350 nm (U.V. Spectrophotometer, JENWAY 6305) [45]. The entrapment efficiency of the drug was calculated by subtracting the amount of un-entrapped drug from the theoretical total drug added that was calculated using the following equation:

Entrapment efficiency (%) = [(AT − AF)/AT] × 100

where AF is the amount of the free un-entrapped colchicine and AT is the total amount of colchicine added.

### 4.4. Vesicular Size and Size Distribution

Size and polydispersity (PDI) of colchicine-loaded niosome vesicle were evaluated by measuring its dynamic light scattering using a Zetasizer apparatus (Malvern Instruments Ltd., Worcestershire, UK). All the experiments were performed in triplicate [46].

### 4.5. Stability Study

Investigating the stability of colchicine-loaded niosome was executed as per the guiding principal of International Conference on Harmonization (ICH). The study was implemented at two different conditions; 4 ± 1 °C and 25 ± 1 °C with humidity 60% for 1 and 3 months in terms of certain parameters, namely; the entrapment efficiency and vesicular size [47].

### 4.6. Preparation of Colchicine Niosomal Emulgel

As stated formerly, for the formulations to be applied properly over the skin, spread simply, and not easily detached from the treated area, it should demonstrate appropriate viscosity. In view of that, the developed niosomal formulation was incorporated into jojoba oil-based emulgel to attain colchicine niosomal emulgel. Primarily, hydrogel base was fabricated by sprinkling specified amount of Na alginate over 10 mL distilled water and mixed well by a magnetic stirrer (Jeio Tech TM-14SB; Medline Scientific, Oxfordshire, UK) until getting a uniform gel base. Next, emulsion was developed by mixing a definite amount of tween 80 with the distilled water for 10 min to form the aqueous phase. The aqueous phase was steadily added to the oily phase (jojoba oil) and the mixture vortexed well for 10 min using classic advanced vortex mixer (VELP Scintifica, Usmate Velate, Italy) until obtaining a consistent homogeneous emulsion. Eventually, emulgel formulation was obtained by mixing both, the hydrogel base with the developed emulsion using a mixer (Heidolph RZR1; Heidolph Instruments, Schwabach, Germany) for 10 min [48]. Table 1 displayed the quantified amount of different independent variables that were used for developing the niosomal formulations along with the values of the observed responses. In order to validate the influence of emulsion and predominantly jojoba oil in further studies, a niosomal gel containing colchicine was fabricated. In which, Na Alginate was added to 20 mL distilled water and stirred well till obtaining a gel, then mixed with the niosomal formulation (5 mL) to get colchicine niosomal gel.

### 4.7. Experimental Design Using BBD

In order to obtain an optimized niosomal emulgel formulation, a matrix of 17 formulas was constructed and fabricated using RSM. BBD is one of the most adequate techniques for analyzing the data and optimizing the formula; therefore, it was employed via constructing three factors, three levels (3^3^) factorial design using Design-Expert version 12.0 software (Stat-Ease, Minneapolis, MN, USA). The factors representing the independent variables were selected as X_1_, X_2_, and X_3_ related to oil concentration, surfactant concentration, and gelling agent concentration, respectively. The influence of the previous factors was studied on the responses R_1_ (viscosity) and R_2_ (in vitro release) of the developed niosomal emulgel. As apparent in Table 6, three different levels (−1, 0, 1) related to lowest, central, and the highest values of examined factors were established alongside their level of variations R_1_ and R_2_ corresponding to the responses. Statistical analysis of the data should be detected via analysis of variance (ANOVA) test. Moreover, correlating the obtained data from the statistical analysis is verified by definite model graphs such as 2D contour and 3D-response surface plots. In addition to a mathematical polynomial equation that could illustrate the response as follows:

R = b_0_ + b_1_X_1_ + b_2_X_2_ + b_3_X_3_ + b_12_X_1_X_2_ + b_13_X_1_X_3_ + b_23_X_2_X_3_ + b_11_X_1_^2^ + b_22_X_2_^2^ + b_33_X_3_^2^

where R represents the detected response, b_0_ represents the intercept; b_1_, b_2_, b_3_, b_12_, b_13_ b_23_, b_11_, b_22_, and b_33_ are the regression coefficients. X_1_, X_2_, and X_3_ signify the main factors; X_1_X_2_, X_1_X_3_, and X_2_X_3_ indicate the interactions between main factors and X_12_, X_22_, and X_32_ point to the polynomial terms.

### 4.8. Characterization

#### 4.8.1. Physical Inspection

Visual inspection of the tested formulations was carried out to examine the physical properties such as color and homogeneity.

#### 4.8.2. Measurement of pH

Formulation’s pH value is very important to ensure safety of the preparation and to avoid any probable skin irritation. Measuring the pH was accomplished using a standardized pH meter (MW802, Milwaukee Instruments, Szeged, Hungary).

#### 4.8.3. Viscosity

The formulations were tested for their viscosities using Brookfield viscometer (DV-II+ Pro, Middleboro, MA, USA) at 25 ± 0.3 °C [49].

#### 4.8.4. Spreadability

This investigation is important for detecting the ability of the formulation to be spread evenly over the affected area by determining the spreadability diameter. In brief, a definite amount of the niosomal gel or emulgel formulation was added in between the two slides. About 0.5 g load was put for 1 min above the slides and the diameter of the spreading area was measured [50].

### 4.9. Assessment of In Vitro Drug Release Studies

The in vitro release investigation of drug from niosomal gel and emulgel preparations was inspected using Franz diffusion system (Logan Instruments Corp., FDC-6, Somerset, NJ, USA). Basically, quantified amounts of each formulation were placed separately over a cellophane membrane (MWCO 2000–15,000) (the donor) that covered the disillusion cell of the apparatus. The system was operated in 7 mL phosphate buffer (the acceptor), pH 7.4 adjusted at 37 ± 0.5 °C and rotated at 50 rpm. Samples were estimated spectrophotometrically at assorted time intervals (0.25, 0.5, 1, 2, 3, 4, 5, and 6 h) at λ_max_ 350 nm. Each experiment was carried out in triplicate.

### 4.10. Release Kinetic Mechanism

Different kinetic models were applied in order to describe the mechanism by which the drug is released from the developed colchicine niosomal gel and emulgel in addition to the correlation coefficient (r2). Plotting drug concentration (C) versus time (t) would provide a profile of the in vitro study. If the model showed a linear plot and displayed the greatest value of (r2), the model is regarded as the best fitting. This study was conducted using the following models [51]:

a. Zero order kinetic C = C0 + kt;

b. First order kinetic C = C0 × ekt;

c. Higuchi kinetic C = k × t_0.5_;

d. Korsmeyer-Peppas kinetic C = k × tn;

where (C) represents the amount of colchicine released in time (t). (C0) indicates the value of (C) at time zero; (k) is the release constant; and (n) indicates the permeation rate exponent [52].

### 4.11. Animal

The animal breeding center at College of Science, King Faisal University provided us with Male Wister rats of about (220 to 250 g). The animals were handled by following the guidelines and ethics approved by Research Ethics Committee (REC) at King Faisal University (KFU-REC/2021-04-17). All animals were maintained in light and dark cycles, accommodated in an organized environment with an ambient temperature (25 ± 2 °C).

### 4.12. Anti-Inflammatory Testing

#### Carrageenan-Induced Rat Paw Edema Test

The action of colchicine as anti-inflammatory drug loaded into different formulations was evaluated via carrageenan-induced rat hind paw edema method. About 0.1 mL of 1% carrageenan in saline was injected subcutaneously into the left hind paw for edema induction, half an hour earlier to drug application [52]. Thirty six male Wister rats were allocated to six groups, each containing five rats of well-adjusted weight, and all groups were induced with inflammation as follows:

Group I: control group that did not receive any treatment.
Group II: treated orally with colchicine solution (1 mg/kg).
Group III: treated with niosomal gel with no drug (placebo I).
Group IV: treated with niosomal emulgel with no drug (placebo II).
Group V: treated with colchicine niosomal gel.
Group VI: treated with colchicine niosomal emulgel (1 mg/kg) [53].

The inflammatory reaction was calculated by assessing the changes in paw thickness using Digital caliper (Electronic digital caliber, AHK, Germany). The percentage of swelling was calculated as follows [54]:

% of swelling = ((V_t_ − V_0_)/V_0_) × 100

where V_0_ symbolizes the thickness of the injected rat hind paw at zero time and V_t_ denotes the thickness after 1, 2, 3, 4, 5, and 12 h of carrageenan injection.

### 4.13. Statistical Analysis

All data were derived from at least three independent experiments, the mean ± standard deviation (SD) from treated groups and that from control group were compared using a one-way analysis of variance (ANOVA) followed by the least significant difference (LSD) as a post-hoc test, using SPSS statistics software, version 14 (IBM Corporation, Armonk, NY, USA). Significance was considered if *p* < 0.05.

## Figures and Tables

**Table 1:** Experimental design for different colchicine niosomal emulgel formulations and their observed values of response.

Formula	Independent Variables			Dependent Response	
	X_1_ (g)	X_2_ (g)	X_3_ (g)	R_1_ (cP)	R_2_ (%)
F1	1	1	1	4772 ± 123	49.5 ± 4.0
F2	1.5	1	0.8	5302 ± 188	42.8 ± 2.6
F3	1.5	0.5	0.8	5645 ± 167	39.2 ± 3.9
F4	1	0.5	1	4553 ± 219	50.8 ± 3.1
F5	1.5	1	1.2	6952 ± 169	28.2 ± 1.2
F6	1.5	0.75	1	6428 ± 179	29.4 ± 2.7
F7	1.5	0.5	1.2	6655 ± 171	29.5 ± 3.5
F8	1	0.75	0.8	4265 ± 184	55.1 ± 3.6
F9	2	1	1	9592 ± 202	22.3 ± 2.5
F10	1.5	0.75	1	5909 ± 246	33.6 ± 3.1
F11	1.5	0.75	1	6125 ± 177	34.2 ± 3.8
F12	2	0.75	1.2	9834 ± 210	21.5 ± 2.7
F13	1.5	0.75	1	6396 ± 268	31.9 ± 2.9
F14	2	0.5	1	8316 ± 176	25.2 ± 2.1
F15	1	0.75	1.2	4991 ± 184	47.2 ± 3.4
F16	2	0.75	0.8	7832 ± 219	26.1 ± 2.3
F17	1.5	0.75	1	6290 ± 237	32.7 ± 3.4

X_1_: oil concentration; X_2_: surfactant concentration; X_3_: gelling agent concentration; R_1_ viscosity; R_2_: in vitro release.

**Table 2:** Results of statistical analysis results for all response.

Source	R_1_		R_2_	
	F-Value	*p*-Value	F-Value	*p*-Value
Model	67.64	<0.0001 *	35.53	<0.0001 *
X_1_	520.34	<0.0001 *	272.19	<0.0001 *
X_2_	3.78	0.0928	0.0850	0.7790
X_3_	52.31	0.0002 *	31.90	0.0008 *
X_1_X_2_	4.03	0.0848	0.1206	0.7386
X_2_X_3_	5.87	0.0459 *	0.5130	0.4970
X_3_X_1_	1.48	0.2638	1.13	0.3229
X_1_^2^	20.80	0.0026 *	10.11	0.0155 *
X_2_^2^	0.0027	0.9600	0.8254	0.3938
X_3_^2^	0.4326	0.5317	1.89	0.2112
Lack of Fit	2.18	0.2333	2.20	0.2304
R^2^ analysis				
R²	0.9886		0.9786	
Adjusted R²	0.9740		0.9510	
Predicted R^2^	0.8805		0.7739	
Adequate Precision	27.6989		20.4175	
Model				
Remark	Quadratic		Quadratic	

X_1_: oil concentration; X_2_: surfactant concentration; X_3_: gelling agent concentration; R_1_: viscosity; R_2_: in vitro release; *: significant.

**Table 3:** Predicted and experimental value of response at optimized conditions.

Response	Predicted Values	Experimental Values
R_1_ (cP)	4187.4 ± 263.4	4598 ± 229.1
R_2_ (%)	55.53 ± 2.34	52.4 ± 3.0

**Table 4:** Characterization of optimized colchicine niosomal gel and emulgel:.

Properties	Colchicine Niosomal Gel	Colchicine Niosomal Emulgel
Visual inspection	Smooth and homogenous	Smooth and homogenous
pH	6.58 ± 0.28	6.73 ± 0.25
Viscosity (cP)	2578.3 ± 214.2	4598 ± 229.1 *
Spreadability (mm)	44.0 ± 1.6	38.3 ± 1.7 *

Values are stated as mean ± (SD) using student *t*-test. * *p* < 0.05 compared to colchicine niosomal gel.

**Table 5:** Release kinetics of various colchicine niosomal formulations.

Formulation	Zero Order Kinetic (r^2^)	First Order Kinetic (r^2^)	Higuchi Kinetic (r^2^)	Korsmeyer-Peppas Kinetic (r^2^)
**Colchicine solution**	0.9370	0.8110	0.9744	0.9341
**Colchicine niosome**	0.8954	0.6800	0.9724	0.9276
**Colchicine niosomal gel**	0.9394	0.8119	0.9867	0.9837
**Colchicine niosomal emulgel**	0.9814	0.9051	0.9856	0.9780

**Table 6:** BBD data displaying independent variables with their level of variation and the examined dependent variables.

Independent Variable	Symbol	Level of Variation		
		Lowest (−1)	Central (0)	Highest (1)
Oil concentration (g)	X_1_	1.0	1.5	2.0
Surfactant concentration (g)	X_2_	0.5	0.75	1.0
Gelling agent concentration (g)	X_3_	0.8	1.0	1.2
**Dependent Variable**	**Symbol**	**Constraints**		
Viscosity (cP)	R_1_	Minimize		
In vitro release (%)	R_2_	Maximize		

## Data Availability

Not applicable.

## References

[1] Patra J.K., Das G., Fraceto L.F., Campos E.V.R., del Pilar Rodriguez-Torres M., Acosta-Torres L.S., Diaz-Torres L.A., Grillo R., Swamy M.K., Sharma S. (2018). Nano based drug delivery systems: Recent developments and future prospects. J. Nanobiotechnol..

[2] Wei Q.-Y., Xu Y.-M., Lau A.T.Y. (2020). Recent Progress of Nanocarrier-Based Therapy for Solid Malignancies. Cancers.

[3] Gharbavi M., Amani J., Kheiri-Manjili H., Danafar H., Sharafi A. (2018). Niosome: A Promising Nanocarrier for Natural Drug Delivery through Blood-Brain Barrier. Adv. Pharmacol. Sci..

[4] Uchegbu L. (2000). Synthetic Surfactant Vesicles: Niosomes and Other Non-Phospholipid Vesicular Systems.

[5] Muzzalupo R., Tavano L. (2015). Niosomal drug delivery for transdermal targeting: Recent advances. Res. Rep. Transdermal Drug Deliv..

[6] Hamishehkar H., Rahimpour Y., Kouhsoltani M. (2012). Niosomes as a propitious carrier for topical drug delivery. Expert Opin. Drug Deliv..

[7] Thakur V., Arora S., Prashar B., Vishal P. (2012). Niosomes and liposomes-vesicular approach towards transdermal drug delivery. Int. J. Pharm. Chem. Sci..

[8] Alhakamy N.A., Aldawsari H.M., Ali J., Gupta D.K., Warsi M.H., Bilgrami A.L., Asfour H.Z., Noor A.O. (2021). Shadab Brucine-loaded transliposomes nanogel for topical delivery in skin cancer: Statistical optimization, in vitro and dermatokinetic evaluation. 3 Biotech.

[9] Sah S.K., Badola A., Nayak B.K. (2017). Emulgel: Magnifying the application of topical drug delivery. Indian J. Pharm. Biol. Res..

[10] Jagdale S., Pawar S. (2017). Gellified Emulsion of Ofloxacin for Transdermal Drug Delivery System. Adv. Pharm. Bull..

[11] Nastiti C.M.R.R., Ponto T., Abd E., Grice J.E., Benson H.A.E., Roberts M.S. (2017). Topical Nano and Microemulsions for Skin Delivery. Pharmaceutics.

[12] Talat M., Zaman M., Khan R., Jamshaid M., Akhtar M., Mirza A. (2021). Emulgel: An effective drug delivery system. Drug Develop. Indust. Pharm..

[13] Sturtevant D., Lu S., Zhou Z.-W., Shen Y., Wang S., Song J.-M., Zhong J., Burks D.J., Yang Z.-Q., Yang Q.-Y. (2020). The genome of jojoba (*Simmondsia chinensis*): A taxonomically isolated species that directs wax ester accumulation in its seeds. Sci. Adv..

[14] Pazyar N., Yaghoobi R., Ghassemi M.R., Kazerouni A., Rafeie E., Jamshydian N. (2013). Jojoba in dermatology: A succinct review. Giornale italiano di dermatologia e venereologia: Organo ufficiale. Soc. Ital. Dermatol. Sifilogr..

[15] Lin T.-K., Zhong L., Santiago J.L. (2017). Anti-Inflammatory and Skin Barrier Repair Effects of Topical Application of Some Plant Oils. Int. J. Mol. Sci..

[16] Meier L., Stange R., Michalsen A., Uehleke B. (2012). Clay Jojoba Oil Facial Mask for Lesioned Skin and Mild Acne—Results of a Prospective, Observational Pilot Study. Forsch. Komplementärmed./Res. Complement. Med..

[17] Gad H.A., Roberts A., Hamzi S.H., Gad H.A., Touiss I., Altyar A.E., Kensara O.A., Ashour M.L. (2021). Jojoba Oil: An Updated Comprehensive Review on Chemistry, Pharmaceutical Uses, and Toxicity. Polymers.

[18] Costa I., Rodrigues R., Almeida F., Favacho H., FalcÃO D., Ferreira A., Vilhena J., Florentino A., Carvalho J.C., Fernandes C. (2014). Development of Jojoba Oil (Simmondsia chinensis (Link) C.K. Schneid.) Based Nanoemulsions. Lat. Am. J. Pharm..

[19] Shahin M., Hady S.A., Hammad M., Mortada N. (2011). Novel Jojoba Oil-Based Emulsion Gel Formulations for Clotrimazole Delivery. AAPS PharmSciTech.

[20] Kim J.H., Kismali G., Gupta S.C. (2018). Natural Products for the Prevention and Treatment of Chronic Inflammatory Diseases: Integrating Traditional Medicine into Modern Chronic Diseases Care. Evid.-Based Complement. Altern. Med..

[21] Richette P., Bardin T. (2010). Colchicine for the treatment of gout. Expert Opin. Pharmacother..

[22] Reyes A.Z., Hu K.A., Teperman J., Wampler Muskardin T.L., Tardif J.-C., Shah B., Pillinger M.H. (2020). Anti-inflammatory therapy for COVID-19 infection: The case for colchicine. Ann. Rheum. Dis..

[23] Gasparyan A.Y., Ayvazyan L., Yessirkepov M., Kitas G. (2015). Colchicine as an anti-inflammatory and cardioprotective agent. Expert Opin. Drug Metab. Toxicol..

[24] Ben-Chetrit E., Bergmann S., Sood R. (2005). Mechanism of the anti-inflammatory effect of colchicine in rheumatic diseases: A possible new outlook through microarray analysis. Rheumatology.

[25] Slobodnick A., Shah B., Krasnokutsky S., Pillinger M.H. (2017). Update on colchicine, 2017. Rheumatology.

[26] Ismail T., Shehata T., Mohamed D., Elsewedy H., Soliman W. (2021). Quality by Design for Development, Optimization and Characterization of Brucine Ethosomal Gel for Skin Cancer Delivery. Molecules.

[27] Sharma A., Singh A.P., Harikumar S.L., Sl H. (2020). Development and optimization of nanoemulsion based gel for enhanced transdermal delivery of nitrendipine using box-behnken statistical design. Drug Dev. Ind. Pharm..

[28] Ammar H.O., Haider M., Ibrahim M., El Hoffy N.M. (2017). In vitro and in vivo investigation for optimization of niosomal ability for sustainment and bioavailability enhancement of diltiazem after nasal administration. Drug Deliv..

[29] Dhoot N.O., Wheatley M.A. (2003). Microencapsulated Liposomes in Controlled Drug Delivery: Strategies to Modulate Drug Release and Eliminate the Burst Effect. J. Pharm. Sci..

[30] Kumar G.P., Rajeshwarrao P. (2011). Nonionic surfactant vesicular systems for effective drug delivery—an overview. Acta Pharm. Sinica B.

[31] Hao Y., Zhao F., Li N., Yang Y., Li K. (2002). Studies on a high encapsulation of colchicine by a niosome system. Int. J. Pharm..

[32] Albash R., El-Nabarawi M.A., Refai H., Abdelbary A.A. (2019). Tailoring of PEGylated bilosomes for promoting the transdermal delivery of olmesartan medoxomil: In-Vitro characterization, ex-vivo permeation and in-vivo assessment. Int. J. Nanomed..

[33] Veronese F.M., Mero A. (2008). The Impact of PEGylation on Biological Therapies. BioDrugs.

[34] Sathyamoorthy N., Magharla D., Chintamaneni P., Vankayalu S. (2017). Optimization of paclitaxel loaded poly (ε-caprolactone) nanoparticles using Box Behnken design. Beni-Suef Univ. J. Basic Appl. Sci..

[35] Bolla P.K., Clark B.A., Juluri A., Cheruvu H.S., Renukuntla J. (2020). Evaluation of Formulation Parameters on Permeation of Ibuprofen from Topical Formulations Using Strat-M® Membrane. Pharmaceutics.

[36] Shehata T.M., Nair A.B., Al-Dhubiab B.E., Shah J., Jacob S., Alhaider I.A., Attimarad M., Elsewedy H.S., Ibrahim M.M. (2020). Vesicular Emulgel Based System for Transdermal Delivery of Insulin: Factorial Design and In Vivo Evaluation. Appl. Sci..

[37] Daood N.M., Jassim Z.E., Gareeb M.M., Zeki H. (2019). Studying the Effect of Different Gelling Agent on the Preparation and Characterization of Metronidazole as Topical Emulgel. Asian J. Pharm. Clin. Res..

[38] Patel J., Patel B., Banwait H., Parmar K., Patel M. (2011). Formulation and evaluation of topical aceclofenac gel using different gelling agent. Int. J. Drug. Dev. Res..

[39] Ibrahim M., Shehata T. (2012). The enhancement of transdermal permeability of water soluble drug by niosome-emulgel combination. J. Drug Deliv. Sci. Technol..

[40] Arora R., Aggarwal G., Harikumar S.L., Kaur K. (2014). Nanoemulsion Based Hydrogel for Enhanced Transdermal Delivery of Ketoprofen. Adv. Pharm..

[41] Agarwal R., Katare O., Vyas S. (2001). Preparation and in vitro evaluation of liposomal/niosomal delivery systems for antipsoriatic drug dithranol. Int. J. Pharm..

[42] Damodharan N. (2020). Mathematical Modelling of Dissolution Kinetics in Dosage forms. Res. J. Pharm. Technol..

[43] Salamanca C.H., Barrera-Ocampo A., Lasso J.C., Camacho N., Yarce C.J. (2018). Franz Diffusion Cell Approach for Pre-Formulation Characterisation of Ketoprofen Semi-Solid Dosage Forms. Pharmaceutics.

[44] Habashy R.R., Abdel-Naim A.B., Khalifa A., Al-Azizi M.M. (2005). Anti-inflammatory effects of jojoba liquid wax in experimental models. Pharmacol. Res..

[45] Abdallah M.H., Elsewedy H.S., AbuLila A.S., Almansour K., Unissa R., Elghamry H.A., Soliman M.S. (2021). Quality by Design for Optimizing a Novel Liposomal Jojoba Oil-Based Emulgel to Ameliorate the Anti-Inflammatory Effect of Brucine. Gels.

[46] Elsewedy H.S., Al Dhubiab B.E., Mahdy M.A., Elnahas H. (2020). Development, optimization, and evaluation of PEGylated brucine-loaded PLGA nanoparticles. Drug Deliv..

[47] Elsewedy H.S., Al-Dhubiab B.E., Mahdy M.A., Elnahas H.M. (2021). Basic Concepts of Nanoemulsion and its Potential application in Pharmaceutical, Cosmeceutical and Nutraceutical fields. Res. J. Pharm. Technol..

[48] Abdallah M.H., Abu Lila A.S., Unissa R., Elsewedy H.S., Elghamry H.A., Soliman M.S. (2021). Preparation, characterization and evaluation of anti-inflammatory and anti-nociceptive effects of brucine-loaded nanoemulgel. Colloids Surf. B Biointerfaces.

[49] Shehata T.M., Khalil H.E., Elsewedy H.S., Soliman W.E. (2021). Myrrh essential oil-based nanolipid formulation for enhancement of the antihyperlipidemic effect of atorvastatin. J. Drug Deliv. Sci. Technol..

[50] Soliman W.E., Shehata T.M., Mohamed M.E., Younis N.S., Elsewedy H.S. (2021). Enhancement of Curcumin Anti-Inflammatory Effect via Formulation into Myrrh Oil-Based Nanoemulgel. Polymers.

[51] Siepmann F. (2013). Mathematical modeling of drug dissolution. Int. J. Pharm..

[52] Shehata T., Ibrahim M., Elsewedy H. (2021). Curcumin Niosomes Prepared from Proniosomal Gels: In Vitro Skin Permeability, Kinetic and In Vivo Studies. Polymers.

[53] El-Feky G.S., El-Naa M.M., Mahmoud A.A. (2019). Flexible nano-sized lipid vesicles for the transdermal delivery of colchicine; in vitro/in vivo investigation. J. Drug Deliv. Sci. Technol..

[54] Ozguney I., Karasulu H.Y., Kantarci G., Sözer S., Güneri T., Ertan G. (2006). Transdermal delivery of diclofenac sodium through rat skin from various formulations. AAPS PharmSciTech.

